# Supplementary material for: Examining Place-Based Neighborhood Factors in a Multisite Peer-Led Healthy Lifestyle Effectiveness Trial for People with Serious Mental Illness
Source: Int J Environ Res Public Health. 2023 Apr 28;20(9):5679. doi: 10.3390/ijerph20095679 (PMC10178706; doi:10.3390/ijerph20095679)
Supplement: Supplementary file 1 [file ijerph-20-05679-s001.zip › ijerph-2272557-supplementary.docx]

Supplementary Materials

*Table S1.* Definitions of Neighborhood level measures

| **Environment construct** | **Variable** | **Type** | **Definition** |
| --- | --- | --- | --- |
| **Social Environment** | | | |
| **Social Environment** | Net population density | Categorical | Number of inhabitants per zip code/total squared kilometers of residential land use in zip code  Categories^a^ are defined as:  1: Low (<20,891.76)  2: Medium (20,891.76 -<58,532.75  3: High (≥58,532.75) |
|  | Age | Categorical | Categories are defined as:  1: Young (<35 years)  2: Middle age (35 -64 years)  3:Old (65+ years) |
|  | Predominant Race | Categorical | Predominant race/ethnicity within the zip code;  If 60% or more of the total population is of a given race/ethnicity it is said to be predominantly of that race/ethnicity, otherwise it is said to be mixed.  Categories are defined as:   \| 0: Mixed \| \| --- \| \| 1: ≥60% white \| \| 2: ≥60% black \| \| 3: ≥60% asian \| \| 4: ≥60% hispanic/latino \| \| 5: ≥60% other race (includes: Native Hawaiian, American Indian/Alaskan Native, and two or more races the other category as defined by the ACS). \| |
|  | Population living in poverty | Categorical | Percentage of population within the zip code that is living in poverty; defined as those whose income is ≤125% of the poverty level and qualify to all Federal Assistantship Programs.  Binary:  0: <50% of the population lives in poverty  1: ≥50% of the population lives in poverty |
|  | Predominant housing type | Categorical | Predominant housing type within the zip code. If 60% or more of the total number of housing units are of a given type it is said to be predominantly of that housing type, otherwise it is said to be mixed  Calculated as: Total number of specific housing type / total housing units within zip code  Categories are defined as:   \| 0: Mixed \| \| --- \| \| 1: ≥60% Single family home \| \| 2: ≥60% row or single fam attached \| \| 3: ≥60% Duplex \| \| 4: ≥60% Apartment (3+ units) \| \| 5: ≥60% Other (includes: Trailer or mobile home and Another type of private home (boat, RV, van, etc.). \| |
|  | Population with a college degree | Categorical | Binary:  0: <60% of the population within the zip code has a college degree  1: 60%+ of the population within the zip code has a college degree |
|  | Housing vacancy in the neighborhood | Categorical | Percentage of vacant units within the zip code.  Categories^a^ are defined as: 1: Low vacancy (<7%)  2: Medium vacancy ( 7-<11.15%)  3: High vacancy (≥11.15%) |
|  | Rented housing units in the neighborhood | Categorical | Percentage of rented (vs owned) units within the zip code.  Categories^a^ are defined as: 1: Low (<43%)  2: Medium ( 43 – < 63.3%)  3: High (≥63.3%) |

^a^Categories defined according to both cities’ quartiles using zip code level data for Philadelphia and New York City

*Table S1.* Definitions of Neighborhood level measures cont.

| **Environment construct** | **Variable** | **Type** | **Definition** |
| --- | --- | --- | --- |
| **Built Environment** | | | |
| **Urban Design** | Land Use Mix | Categorical | −[Σi (pi)*(ln pi)]/(ln k) where p = proportion of total land uses, i = land use category, ln = natural logarithm, k = number of land uses. Range is 0 to 1.  Categories^a^ are defined as:  1: Low (< .67)  2: Medium (.67 – < .82)  3: High (≥ .82) |
|  | Connectivity | Categorical | Number of 3-to-4-way intersections per zip code area / total zip code area in squared kilometers.  Categories^a^ are defined as:  1: Low (<269)  2: Medium (269 – <363.7)  3: High (≥363.7) |
|  | Walkability Index | Categorical | z-scored net population density + 2(z-scored connectivity) + z-scored land-use mix ^1^  Categories^a^ are defined as:  1: Low (< .37)  2: Medium (.37 – <1.41)  3: High (≥1.41) |
|  | Transit Stops Density | Categorical | Total number of all types of transit stops / total zip code area in squared kilometers.  Categories^a^ are defined as:  1: Low (<22.08)  2: Medium (22.08 – <36.45)  3: High (≥36.45) |

^a^Categories defined according to both cities’ quartiles using zip code level data for Philadelphia and New York City

*Table S1.* Definitions of Neighborhood level measures cont.

| **Environment construct** | **Variable** | **Type** | **Definition** |
| --- | --- | --- | --- |
| **Health Care Environment** | Hospital Density | Categorical | Number of Hospitals per zip code area/ total population living within the zip code  Categories^a^ are defined as:  1: Low (0)  2: Medium (>0 – <.000055)  3: High (≥.000055) |
|  | Pharmacy and drug stores density | Categorical | Number of drug stores or pharmacies per zip code area/ total population living within the zip code  Categories^a^ are defined as:  1: Low (<.00014)  2: Medium (.00014 – <.0003)  3: High (≥.0003) |
| **Legal Drug Environment** | Licensed tobacco sale places density | Categorical | Number of Licensed tobacco places per zip code area/ total population living within the zip code  Categories^a^ are defined as:  1: Low (0)  2: Medium (>0 – <.00002)  3: High (≥.00002) |
|  | Liquor store density | Categorical | Number of Liquor stores per zip code area/ total population living within the zip code  Categories^a^ are defined as:  1: Low (<.0001)  2: Medium (.0001– <.0002)  3: High (≥.0002) |

^a^Categories defined according to both cities’ quartiles using zip code level data for Philadelphia and New York City

*Table S1.* Definitions of Neighborhood level measures cont.

| **Environment construct** | **Variable** | **Type** | **Definition** |
| --- | --- | --- | --- |
| **Physical Activity Environment** | *Public and private recreation facilities* | | |
|  | Public Parks density | Categorical | Total Park area within the zip code in square kilometers / total population living within the zip code  Categories^a^ are defined as:  1: Low (<.000211)  2: Medium (.000211 - <.00632)  3: High (≥.00632) |
|  | Public Recreation centres density | Categorical | Number of Public and Community Recreational Centers/ total population living within the zip code  Categories^a^ are defined as:  1: Low (<.00002)  2: Medium (.00002- <.00005)  3: High (≥.00005) |
|  | YMCA centres density | Categorical | Number of YMCAs within the zip code area / total population living within the zip code  Categories^a^ are defined as:  1: Low (0)  2: Medium (NA)  3: High(>0) |
|  | Private fitness and recreation sport centres density | Categorical | Number of Private fitness and recreational sports centres per zip code/ total population living within the zip code  Categories^a^ are defined as:  1: Low (<.17)  2: Medium (.17- <1.56)  3: High (≥01.56) |
|  | *Walking and cycling infrastructure* | | |
|  | Sidewalk coverage | Categorical | total sidewalk length (linear kms)/total linear kilometers of roads within the zip code  Categories^a^ are defined as:  1: Low (<1.11)  2: Medium (1.11 - < 1.334)  3: High (≥1.334) |
|  | Bicycle lanes coverage | Categorical | total bike lane length (linear kms)/total linear kilometers of roads within the zip code  Categories^a^ are defined as:  1: Low (<.05)  2: Medium (.05 – .12)  3: High (≥.12) |
|  | Protected bicycle lanes coverage | Categorical | Total protected bike lane length (linear kms)/total linear kms of roads within the zip code  Categories^a^ are defined as:  1: Low (<.007)  2: Medium (.007 - < 0.27)  3: High (≥0.27) |

^a^Categories defined according to both cities’ quartiles using zip code level data for Philadelphia and New York City

*Table S1.* Definitions of Neighborhood level measures cont.

| **Environment construct** | **Variable** | **Type** | **Definition** |
| --- | --- | --- | --- |
| **Food Environment** | *Food Access Points* | | |
|  | Supermarket and grocery stores density | Categorical | Number of supermarket and grocery stores per zip code area/ total population living within the zip code  Categories^a^ are defined as:  1: Low (< .67)  2: Medium (.67 - < 1.11)  3: High (≥1.11) |
|  | Bodegas, convenience and general stores density | Categorical | Number of convenience stores/general store per zip code area/ total population living within the zip code  Categories^a^ are defined as:  1: Low (<.08)  2: Medium (.08 - < .15)  3: High (≥ .15) |
|  | *Food Service Points* | | |
|  | Fast Food restaurant density | Categorical | Number of Fast-Food restaurants per zip code area/ total population living within the zip code  Categories^a^ are defined as:  1: Low (<.40)  2: Medium ( .40- < .61)  3: High (≥ .61) |

^a^Categories defined according to both cities’ quartiles using zip code level data for Philadelphia and New York City

*Table S2.* Definitions of Individual-level dietary and physical activity behavior measures.

| **Variable** | **Type** | **Definition** |
| --- | --- | --- |
| Less than 5 Portions of Fruits and Vegetables per day | Categorical | Binary:  0: Participant reported eating 5 or more portions per day  1: Participant reported eating less than 5 portions per day |
| 1 or more Portions of SSB per day | Categorical | Binary:  0: Participant reported drinking less than 1portions per day of sugar sweetened beverages  1: Participant reported drinking 1 or more portions per day of sugar sweetened beverages |
| Less than 150 minutes of walking per week | Categorical | Binary:  0: Participant reported 150 minutes or more of walking per week  1: Participant reported less than 150 minutes of walking per week |
| Less than 150 minutes of MVPA per week | Categorical | Binary:  0: Participant reported 150 minutes or more of moderate-to-vigorous physical activity per week (excluding walking)  1: Participant reported less than 150 minutes of moderate-to-vigorous physical activity per week (excluding walking) |

**References**

Frank LD, Sallis JF, Saelens BE, et al. The development of a walkability index: application to the Neighborhood Quality of Life Study. *Br J Sports Med*. Oct 2010;44(13):924-33. doi:10.1136/bjsm.2009.058701
